# Inhibition of the miR-192/215–Rab11-FIP2 axis suppresses human gastric cancer progression

**DOI:** 10.1038/s41419-018-0785-5

**Published:** 2018-07-13

**Authors:** Xiaojing Zhang, Yin Peng, Yong Huang, Shiqi Deng, Xianling Feng, Gangqiang Hou, Huijuan Lin, Jian Wang, Ruibin Yan, Yanqiu Zhao, Xinmin Fan, Stephen J. Meltzer, Song Li, Zhe Jin

**Affiliations:** 1 Department of Pathology, School of Basic Medical Sciences, Guangdong Key Laboratory for Genome Stability & Disease Prevention, Shenzhen Key Laboratory of Micromolecule Innovatal Drugs, Shenzhen University Health Sciences Center, Shenzhen, Guangdong People’s Republic of China; 2 Guangdong Province Key Laboratory of Molecular Oncologic Pathology, Guangzhou, Guangdong People’s Republic of China; 3 Department of Medical Image Center, Nanshan Hospital, Guangdong Medical College, Shenzhen, Guangdong Province People’s Republic of China; 4 Department of Pathology and Pathophysiology, The Guangzhou Medical University, Guangzhou, Guangdong People’s Republic of China; 5 Laboratory of Chemical Genomics, The Shenzhen Graduate School of Peking University, Shenzhen, Guangdong People’s Republic of China; 6 Department of Medicine/GI Division, Johns Hopkins University and Sidney Kimmel Cancer Center, Baltimore, MD USA

## Abstract

Less than a century ago, gastric cancer (GC) was the most common cancer throughout the world. Despite advances in surgical, chemotherapeutic, and radiotherapeutic treatment, GC remains the number 3 cancer killer worldwide. This fact highlights the need for better diagnostic biomarkers and more effective therapeutic targets. RAB11-FIP2, a member of the Rab11 family of interacting proteins, exhibits potential tumor suppressor function. However, involvement of RAB11-FIP2 in gastric carcinogenesis is yet to be elucidated. In this study, we demonstrated that RAB11-FIP2 was downregulated in GC tissues and constituted a target of the known onco-miRs, miR-192/215. We also showed that functionally, Rab11-FIP2 regulation by miR-192/215 is involved in GC-related biological activities. Finally, RAB11-FIP2 inhibition by miR-192/215 affected the establishment of cell polarity and tight junction formation in GC cells. In summary, this miR-192/215–Rab11-FIP2 axis appears to represent a new molecular mechanism underlying GC progression, while supplying a promising avenue of further research into diagnosis and therapy of GC.

## Introduction

Gastric cancer (GC) is the third-most common cause of cancer death worldwide, there are approximately 951,600 new GC cases and 723,100 deaths every year^1^. However, despite recent progress in the detection and treatment of early GC, the prognosis of this disease remains quite poor^2,3^. A better understanding of the molecular pathogenesis of GC, along with more effective targeted therapies, is therefore necessary. Therefore, we focus on discovering novel, dependable, and non-invasive biomarkers of GC.

The Rab11-family interacting proteins (Rab11-FIPs), which comprise at least six mammalian genes, Rip11, Rab11-FIP1, Rab11-FIP2, Rab11-FIP3, RCP, and Rab11-FIP4, are well-documented participants in the regulation of apical membrane recycling and transcytosis in epithelial cells^4^. Rab11-family interacting protein 2 (Rab11-FIP2) forms a ternary complex with Rab11 and the motor protein myosin Vb to regulate basolateral-to-apical transcytosis in MDCK(Madin-Darby canine kidney) cells^5,6^. The complex of Rab11-FIP2/Rab11a/myosin Vb participates in Rab11-mediated recycling pathways^5^. Naslavsky et al.^7^ showed that Rab11-FIP2 and Eps15 homology domain (EHD) 1 acted in a coordinated fashion to mediate early endocytic recycling. To date, emerging evidence shows that Rab11-FIPs are involved in tumor progression and metastasis. However, the participation of Rab11-FIP2 in human gastric carcinogenesis remains unclear. MicroRNAs (miRs) are intimately involved in tumorigenesis, acting either as oncogenes or tumor suppressor genes^8^. Alterations in miR expression have been observed in GC, suggesting that miR dysfunction contributes to gastric tumorigenesis and progression. In this study, Rab11-FIP2 was found to be a target of miR-192/215, previously identified as gastric oncomiRs^9^. We then further explored the involvement of the miR-192/215–Rab11-FIP2 axis in gastric carcinogenesis.

Herein, we demonstrate that Rab11-FIP2 displays decreased mRNA and protein expression in GC, and that the miR-192/215–Rab11-FIP2 axis regulates GC cell proliferation, migration, and invasion. We also show that cell junction and polarity are involved in GC-related biological activities of Rab11-FIP2. Moreover, we demonstrate that Rab11-FIP2 dysregulation is associated with lymphatic metastasis in GC patients. Taken together, these findings provide an experimental basis for investigating miR-192/215–Rab11-FIP2 axis as a potential therapeutic target in GC.

## Results

### Decreased expression and potential tumor-suppressive function of Rab11- FIP2 in GC

**Fig. 1:**
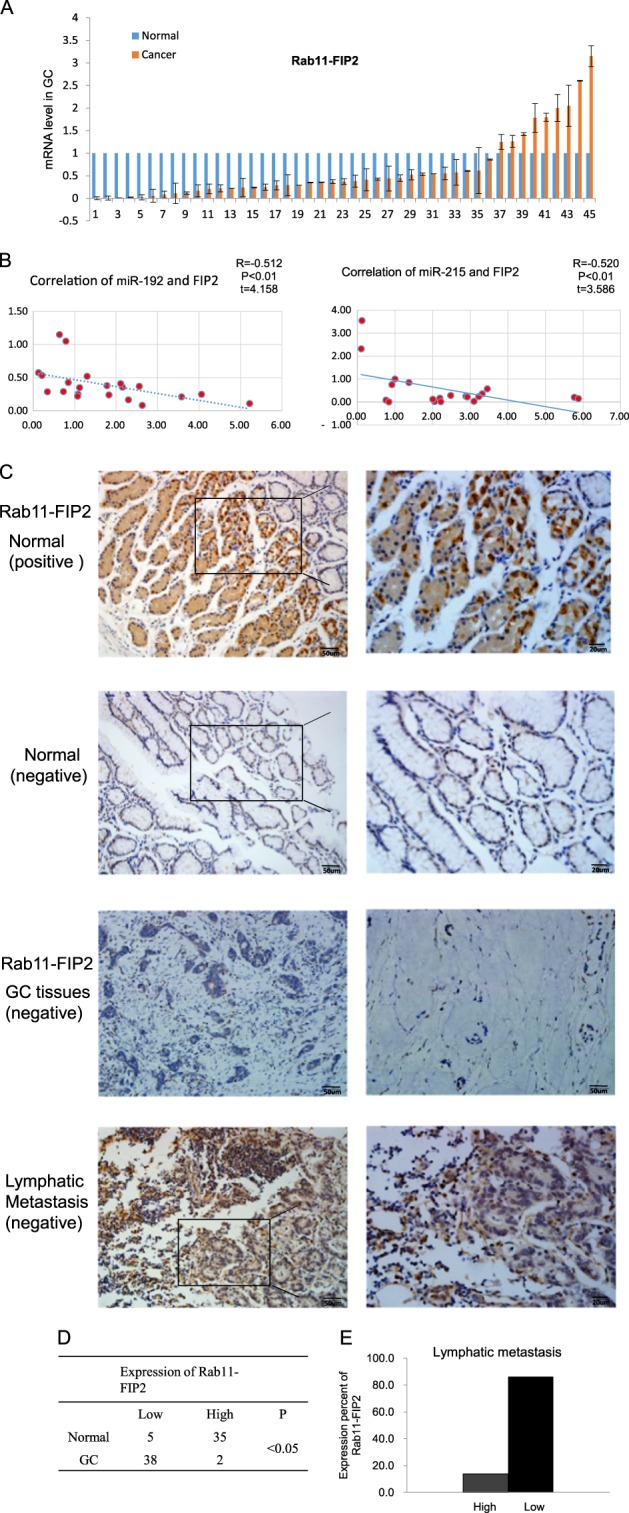
Expression of Rab11-FIP2 is low in GC tissues. **a** RNA levels of Rab11-FIP2 in 45 pairs of GC tissues were determined using RT-PCR. **b** Correlation analysis of miR-192/215 and Rab11-FIP2 RNA levels in 21 paired GC tissues. **c** The protein levels of Rab11-FIP2 were tested by IHC in tissues microarray. **d** Statistical data of Rab11-FIP2 proteins by IHC in GC tissues. **e** Expression percentage of Rab11-FIP2 proteins by IHC in lymphatic metastatic tissues

Expression levels of Rab11-FIP2 were measured in 45 paired tumor tissue specimens from GC patients by real-time reverse transcription polymerase chain reaction (RT-PCR). Among these 45 paired specimens, only nine showed overexpression of Rab11-FIP2 mRNA in cancer vs. normal tissues. Overall, mRNA levels of Rab11-FIP2 were significantly lower in cancers than in matched normal tissues (Fig. 1a). Additionally, paired analysis of 21 paired tissues showed an inverse correlation between miR-192/215 and RAB11-FIP2 levels (*R* = −0.512, *p* < 0.01, *t* = 4.158; *R* = −0.520, *p* < 0.01, *t* = 3.586, respectively; Fig. 1b). Next, Rab11-FIP2 protein expression levels were assayed by immunohistochemistry (IHC) in a GC tissue microarray. This microarray consisted of 40 GC cases including primary tumors, normal tissues, and metastatic or non-metastatic lymph node tissues. Compared with normal tissues, Rab11-FIP2 protein was significantly lower in cancer tissues (Fig. 1c, d). Thirty-five (87.5%) of 40 normal mucosae exhibited high levels of Rab11-FIP2 protein, while only two (5%) GC specimens expressed abundant Rab11-FIP2 (*p* < 0.005). To investigate the involvement of Rab11-FIP2 in GC metastasis, we analyzed Rab11-FIP2 expression in metastatic lymph nodes. Among 29 cases with metastatic lymph nodes, 86.2% (25) showed reduced expression of Rab11-FIP2, and expression levels were high in only 13.8% (4/29) metastatic lymph nodes (Fig. 1e). There were no significant correlations between RAB11-FIP2 expression and age, gender, differentiation, or other clinical parameters (Supplementary Table 2). A significant difference in RAB11-FIP2 expression was found between normal and GC tissues using the Rank Sum Test, with expression being lower in GC tissues. Meanwhile, Rab11-FIP2 levels also declined in lymphatic metastatic tissues compared with normal mucosae (Table 1). These findings support the notion that Rab11-FIP2 functions as a tumor suppressor in GC.

**Table 1:** Expression levels of Rab11-FIP2 by IHC in GC, lymphatic metastatic, and adjacent normal tissues

Group	FIP2 expression				Total	Mean rank
	−	+	+ +	+ + +		
Normal mucosa	4	1	16	19	40	86.60
GC tissues	36	2	2	0	40	36.25
Lymphatic metastatic tissues	23	2	4	0	29	44.19

Results of immunohistochemical staining; *χ*^2^ = 67.443

*p* < 0.001 for difference among the three groups

### Rab11-FIP2 is a target of miR-192/215 in GC

**Fig. 2:**
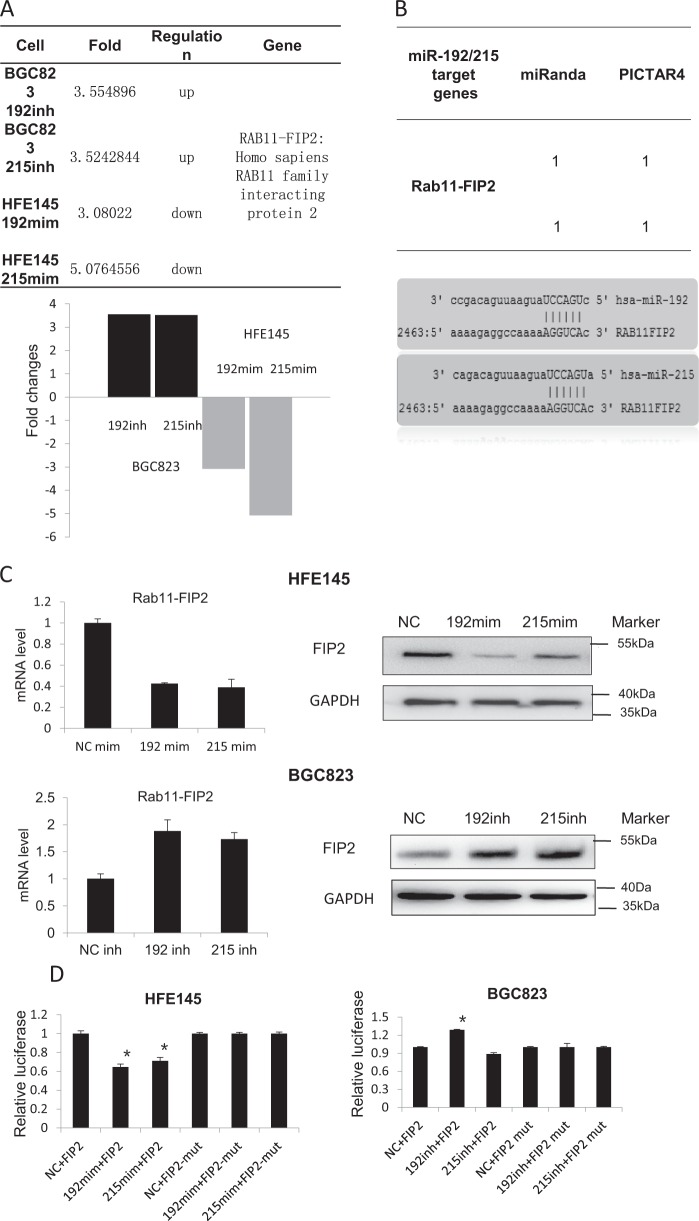
Rab11-FIP2 is the target of miR-192 /215. **a** Rab11-FIP2 is the target of miR-192/215 indicated by gene array. Each sample had been replicated by three probs. **b** Targets of miR-192/215 were predicted based on the database information. **c** The regulation of Rab11-FIP2 by miR-192/215 was verified by Western blot assays. **d** Rab11-FIP2 3′ UTR luciferase assays in HFE145 cells transfected with miR-192/215 mimics and in BGC823 cells transfected with miR-192/215 inhibitors. NC: negative control of miR; 192: miR-192; 215: miR-215; FIP2: Rab11-FIP2 3′ UTR; mim: mimic; inh: inhibitor; mut: mutation. * Means statistical significance

In our previous study, we found miRs-192/215 were expressed at high levels in BGC823 GC cells but at low levels in immortalized normal HFE145 cells (Supplementary Figure 1). In order to discover additional targets of miR-192/215 that were significantly dysregulated in GC, custom microarray analyses with an expanded set of probes were performed. HFE145 and BGC823 cells were transfected with mimics or inhibitors of miR-192 or -215 or a negative control (NC) miR. Results of these analyses revealed that Rab11-FIP2 was upregulated 3.5-fold in BGC823 cells transfected with both miR-192 and miR-215 inhibitors vs. NC. In contrast, Rab11-FIP2 was downregulated 3- or 5-fold in HFE145 cells transfected with miR-192 or -215 mimics, respectively (Fig. 2a). In silico searches showed that a “seed region” binding site existed between Rab11-FIP2 and the 3′UTRs of both miRs-192 and -215 (Fig. 2b). In addition, other genes were dysregulated by miR-192/215, as shown in Supplementary Table 3. To validate the results of microarray and in silico searches, mRNA and protein levels of Rab11-FIP2 were individually measured. mRNA levels showed that Rab11-FIP2 was decreased 2.4 or 2.6-folds by miR-192 or -215 mimics, respectively. In contrast, Rab11-FIP2 was increased 1.88- or 1.73-fold by miR-192 or -215 inhibitors, respectively. Western blotting confirmed that protein levels of Rab11-FIP2 were induced by inhibitors and suppressed by mimics of both miR-192 and miR-215 in gastric cells. (Fig. 2c). Finally, luciferase reporter assays were performed to validate direct action of these two miRs on RAB11-FIP2. Relative to NC, transient transfection of the wild-type RAB11-FIP2-luc reporter with miR-192 mimics into HFE145 cells showed significantly decreased luciferase activity (*p* < 0.05). Similarly, in BGC823 cells transfected with inhibitors of miR-192 or miR-215, luciferase activity was markedly higher (*p* < 0.05) (Fig. 2d). There was no change in luciferase activity in HFE145 cells transfected with miR-215 mimics, possibly due to cell line idiosyncrasy. In summary, Rab11-FIP2 was proven to constitute a target of miR-192/215 and to be negatively regulated by miR-192/215 in GC and immortalized normal cells.

### Rab11-FIP2 is involved in miR-192- and 215-induced progression of GC

**Fig. 3:**
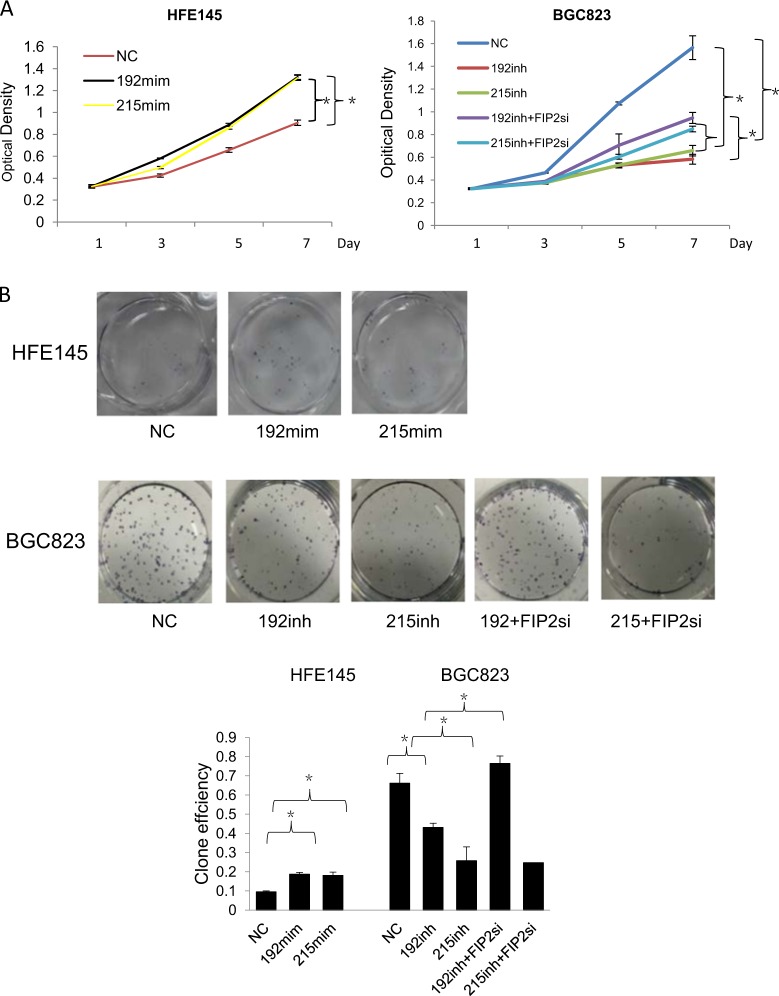

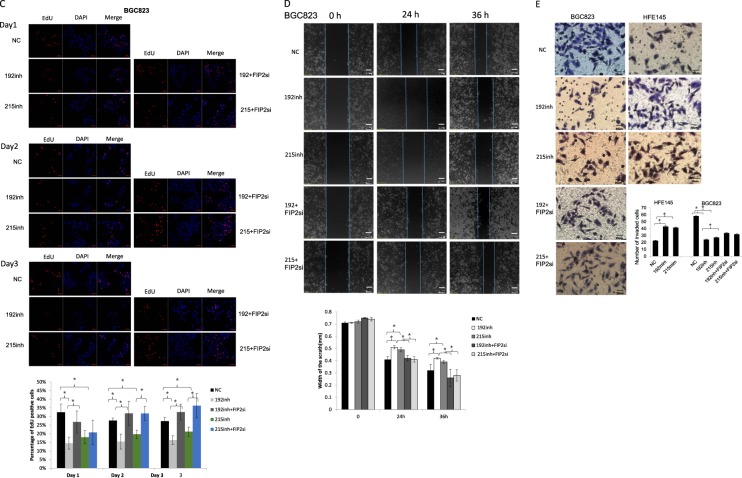
miR-192/215–Rab11-FIP2 axis affected cell growth and invasion of BGC823 and HFE145. **a**, **b**, **c** Effect of miR-192/215 targeting Rab11-FIP2 on cell proliferation by CCK8 assays, colony formation assays, and EdU assays. The numbers of colonies containing more than 50 cells were counted. Data represent mean ± SD. The cell proliferation was examined by EdU incorporation assay at 24 h after transfection. The detection was performed at 24 h, 48 h, and 72 h. The data are reported as mean ± SD for three independent experiments. **d** Effect of miR-192/215 targeting Rab11-FIP2 on cell scratch assays. **e** Effect of miR-192/215 targeting Rab11-FIP2 on cell invasion by Boyden chamber. Morphologic comparison of cells penetrating the artificial basement membrane was also shown. Data represent mean ± SD. NC: negative control of miR; 192: miR-192; 215: miR-215; FIP2Si: siRNA Rab11-FIP2; mim: mimic; inh: inhibitor. * Means statistical significance

To explore the effects on proliferative properties of miR-192/215 mediated by RAB11-FIP2 in GC, we performed functional assays in vitro and in vivo. Mediation of anti-proliferative effects of RAB11-FIP2 by miR-192/215 were confirmed by MTT and colony formation assays. Proliferation of cells was increased by miR-192/215 mimics in HFE145 cells (*p* < 0.05; Fig. 3a, left). In contrast, miR-192/215 inhibition in BGC823 cells led to significant decreases in growth rates, and a Rab11-FIP2 siRNA rescued this inhibition of proliferation (*p* < 0.05; Fig. 3a, right). In agreement with MTT results, colony formation assays confirmed mediation of the anti-proliferative effects Rab11-FIP2 by miR-192/215 (Fig. 3b). EdU assays showed that the transfection of miR-192/215 inhibitor in BGC823 resulted in the decrease of cell proliferation relative to NC (miR-NC, *p* < 0.01). Transfection of siRNA of Rab11-FIP2 can reverse the inhibition induced by miR-192/215 inhibitors (*p* < 0.01, Fig. 3c). Interestingly, revealed by the time kinetic assays, the changes were more visible at later time points. At the 24 h, there is no rescue effect in the BGC823 cells transfected with siRNA of Rab11-FIP2. However, at the following time point of 48 h and 72 h, the cell proliferation rescued was significantly increased. Next, wound healing and transwell assays were done to investigate the effects of Rab11-FIP2 on cell migration and invasion. Notably, inhibition of miR-192/215 suppressed cell migration and invasion compared with control (*p* < 0.05, Fig. 3d, e) in BGC823 cells. We then co-transfected a miR-192 or -215 inhibitor and a RAB11-FIP2 siRNA into BGC823 cells. The Rab11-FIP2 siRNA significantly stimulated motility and invasiveness of BGC823 cells treated with miR-192 or -215 inhibitors (Fig. 3d, e). In the scratch assay, we also conducted the experiment at different time points. We found that the migration ability was increased at later time point. Taken together, these findings revealed that regulation of Rab11-FIP2 by miR-192/215 is involved in the malignant phenotype of GC.

**Fig. 4:**
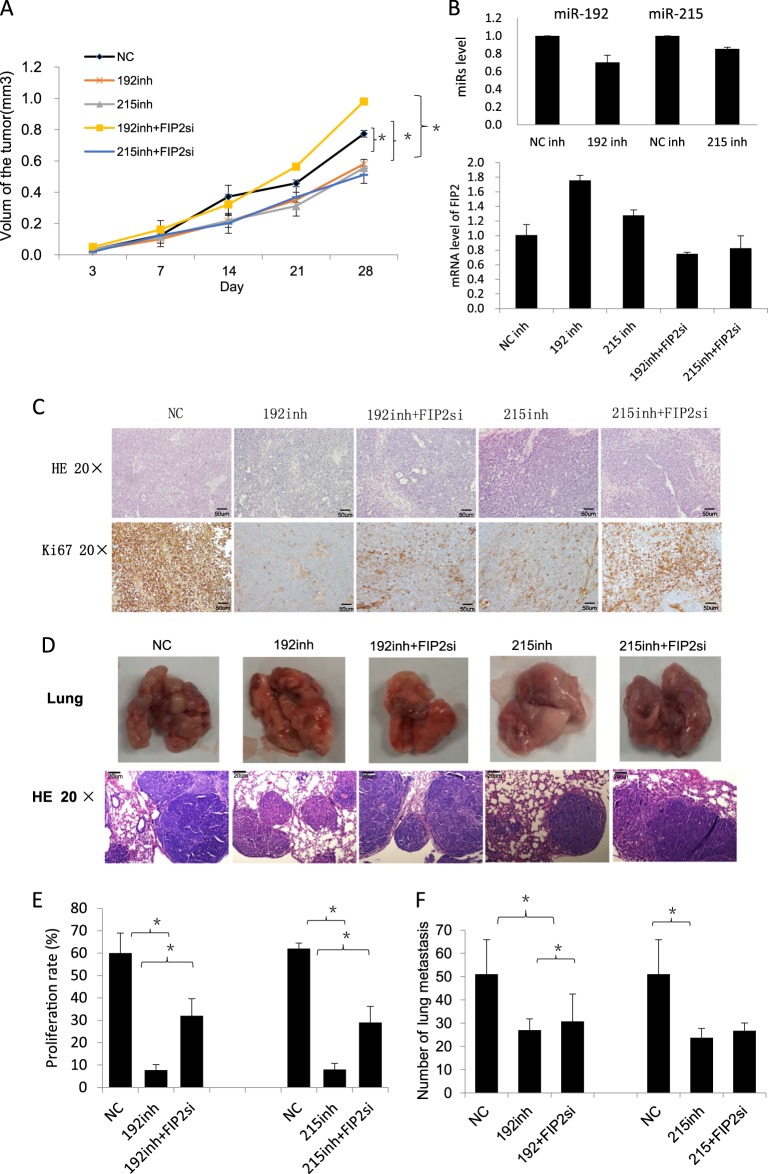
Inhibition of miR-192/215–Rab11-FIP2 axis suppressed the invasion and metastasis of GC in vivo. **a** Assays of subcutaneous tumor growth in mice. Subcutaneous tumor volume was analyzed by repeated measurements. **b** Test of the efficiency of miRs inhibitors and Rab11-FIP2 siRNAs. **c** Assays of subcutaneous tumor staining with H&E and Ki67. **d** Assays of lung metastasis in mice. The number of metastatic lung nodules was counted. **e** Statistical data of Ki67 staining. **f** Statistical data of metastasis lesions of lung tissues. NC: negative control of miR; 192: miR-192; 215: miR-215; FIP2si: SiRNA Rab11-FIP2; mim: mimic; inh: inhibitor; * Means statistical significance

The above effects of Rab11-FIP2 on GC cell proliferation were subsequently confirmed in vivo by subcutaneous tumor xenograft assays in nude mice. Treatment of BGC823 cells with miR-192 or -215 inhibitors resulted in decreased xenograft growth compared with an NC group (Fig. 4a). In contrast, BGC823 cells treated with an miR-192 inhibitor and co-transfected with a Rab11-FIP2 siRNA restored rapid tumor growth, while there was no appreciable increase in cells treated with an miR-215 inhibitor. Next, the effectiveness of the Rab11-FIP2 siRNA and the miR inhibitors was validated by RT-PCR. In tumor xenografts, miR-192/215 levels were decreased by both inhibitors, while Rab11-FIP2 mRNA levels were decreased by the siRNA (Fig. 4b). To assess proliferation effects in xenograft experiments, Ki67 staining was performed. This assay revealed that Ki67 expression was lower in miR-192/215 inhibitor-treated cases, but markedly higher in miR-192/215 inhibitor/siRab11-FIP2 co-treated tumors (Fig. 4c). Proliferation of miR-192/215 inhibitor-treated tumors were reduced significantly, whereas siRNA Rab11-FIP2 increased proliferation (Fig. 4e). Assays of cell apoptosis were then carried out by TUNEL; there were no significant differences in apoptosis among the different treatment conditions. Thus, the miR-192/215–Rab11-FIP2 axis appeared to have no effect on apoptosis (Supplementary Figure 3).

To evaluate the effects of miRs-192 and -215 on tumor metastasis in vivo, BGC823 cells transfected with miR-192 or -215 inhibitors and Rab11-FIP2 siRNA were injected into the tail vein of nude mice. BGC823/miR-192/215 inhibitors attenuated the development of metastasis compared with the NC group, and effect which was rescued by the miR-192/Rab11-FIP2 siRNA combination (Fig. 4d–f). Taken together, these results supported the conclusion that regulation of Rab11-FIP2 by miR-192/215 contributes to the proliferation and metastasis of GC cells in vivo, supporting the importance of the miR-192/Rab11-FIP2 axis in this malignancy.

### Regulation of Rab11-FIP2 by miR-192/215 is involved in cell junction formation and epithelial–mesenchymal transition (EMT)

**Fig. 5:**
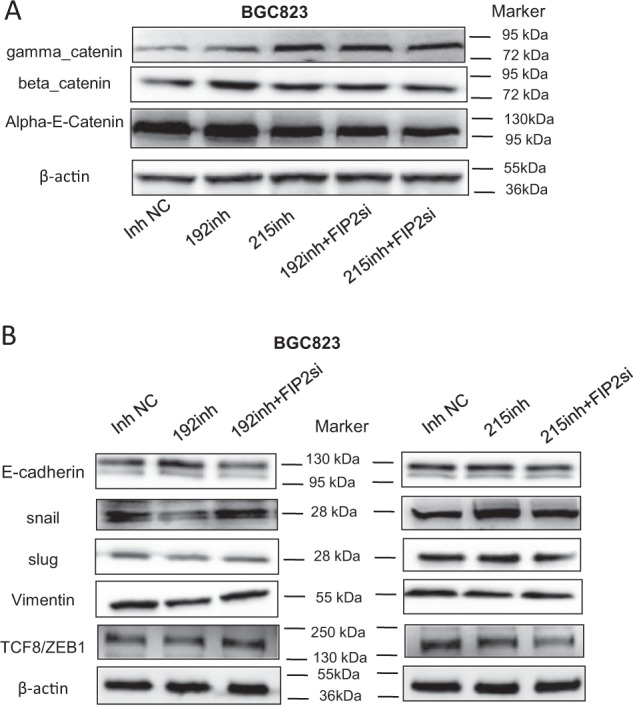
miR-192/215 targeting Rab11-FIP2 primed EMT through adherens junction . **a** Adherent junction proteins expression. **b** EMT proteins expression. NC: negative control of miR; 192: miR-192; 215: miR-215; FIP2si: siRNA Rab11-FIP2; mim: mimic; inh: inhibitor

While the above data demonstrated an association between the regulation of Rab11-FIP2 by miR-192/215 and GC development or progression, the mechanism underlying this relationship remained unclear. Therefore, we sought to clarify this mechanism. Rab11-FIP2 is known to be involved in adhesion between epithelial cells^10^, so we examined adherence junction proteins in this context. To validate transfection efficiency, we measured the expression of the regulation of several adherence proteins by miR-192/215 and an siRNA against Rab11-FIP2 (Supplementary Figure 2). Inhibition of miR-192/215 increased the expression of α-catenin, β-catenin, and γ-catenin, while Rab11-FIP2 siRNA decreased these protein levels (with the exception of the combined miR-215 inhibitor/Rab11-FIP2 siRNA group; Fig. 5a). This result suggests that Rab11-FIP2 is involved in adherence junction dysregulation during GC development or progression. It is known that junctions between epithelial cells become flexible, leading to cancer development and metastasis^11^.

Next, we investigated EMT-related proteins. Relative to the NC group, Snail, Slug, Vimentin, and TCF8 were decreased by inhibition of miR-192, which in turn upregulated Rab11-FIP2. After inhibition of Rab11-FIP2 by an siRNA, the expression levels of these proteins were rescued. However, the effect of miR-215 was not clear, nor was the rescue of miR-215 inhibition by the siRNA (Fig. 5b). After inhibiting miR-192/215, E-cadherin levels were increased in BGC823 cells. Conversely, when Rab11-FIP2 was inhibited by siRNA, E-cadherin levels were reduced. For other markers of EMT, including ZO1 and Claudin, there were no effects on protein levels induced by exogenous regulation of miR-192/215 (Supplementary Figure 4). These results confirmed that regulation of Rab11-FIP2 by miR-192/215 is involved in cell junction and EMT.

## Discussion

This study establishes that Rab11-FIP2 is downregulated at both the mRNA and protein levels in GC tissues and constitutes a direct target of miRs-192 and -/215. Under the regulation of miR-192/215, Rab11-FIP2 affects cancer-relevant biological properties of GC cells. For example, inhibition of miR-192/215 inhibited the growth of GC cells, whereas inhibition of Rab11-FIP2 by siRNA rescues this growth inhibition. Similarly, migration and invasion were modified by regulation of Rab11-FIP2, both in vitro and in vivo. These results suggest that Rab11-FIP2 is a gastric tumor suppressor under the regulation of miR-192/215.

Rab11-FIP2 is a member of the Rab11-binding protein family, with association with Rab11a and/or myosin Vb controlling membrane trafficking^5^. This gene has been implicated in endosome recycling as well as receptor-mediated endocytosis^10^. Rab11-FIP2 is a crucial substrate for MARK2 action, being phosphorylated on its Ser-227 by MARK2, which contributes to cellular polarity establishment^12^. Therefore, increasing evidence suggests the involvement of Rab11-FIP2 in establishment of polarity in epithelia^11,13–15^. Lapierre et al.^12,16^ showed that MDCK cells transfected with MARK2 shRNA exhibited decreased phosphorylation of serine 277 of Rab11-FIP2 and retarded establishment of cell polarity. Additional studies demonstrated that phosphorylation of Rab11-FIP2 on Ser-227 was a crucial event for both the establishment of epithelial polarity and the proper formation of cellular junctions^16^. Cell polarity and cellular junctions are both key steps in tumorigenesis, invasion, and metastasis^17^. For example, Nam^18^ investigated the epithelial cell polarity protein Lgl2 during GC progression, finding that Lgl2 loss occurs at an early stage of gastric carcinogenesis and contributes to GC progression. There are two key roles for tumor suppression by apical–basal polarity in epithelial cells: regulation of asymmetric cell division and maintenance of the apical junctional complex^19^. Tumors of epithelial origin lose these characteristics as they progress from benign growth to malignant carcinoma, and this loss is associated with a poor clinical prognosis^17,20^. For this reason, in addition to bioinformatic bases, studies of Rab11-FIP2 in GC were launched in the current manuscript.

As a result of these studies, Rab11-FIP2 was predicted and identified as a new target of miR-192/215 in GC (through gene microarray and in silico searches). We discovered that Rab11-FIP2 was expressed at very low levels in cancer vs. normal tissues. In lymphatic metastases, Rab11-FIP2 was also expressed at low levels. Previously, to our knowledge, only two papers tested expression of Rab11-FIP2 in tumors. Xu et al.^21^ suggested that Rab11-FIP2 was upregulated in CRC vs. peritumoral tissues by Oncomine data-mining analyses, Western blotting, and IHC. Dong^22^ investigated the expression of Rab11-FIP2 by IHC in 86 GC patients. However, these researchers showed that expression of Rab11-FIP2 was significantly elevated in GC. In their studies, a total IHC score was calculated by adding nuclear and cytoplasmic scores. In contrast, we calculated a positive score for Rab11-FIP2 based on the percent positivity of stained tumor cells (0–4) and their staining intensity (0–3). In addition, clinical, molecular, and pathological data suggest that GC exhibits a high degree of molecular heterogeneity^23,24^. Several studies have reported that there are molecular differences between the intestinal and diffuse types of GC^23,25^. For example, a high rate of RHOA mutations occur in the Lauren diffuse-type but not in intestinal-type GC^24,26^. In our assays, most of the GCs studied were diffuse-type adenocarcinomas. The histological subtypes of GC in Dong’s study^22^ were not clear, but representative figures for IHC were intestinal-type GCs. Thus, the differences of GC subtype may partially result from expression heterogeneity. On the other hand, different antibodies used in our vs. Dong’s study may have given rise to different expression results, due to unique immunogenic sequences. Furthermore, results of qRT-PCR were consistent with tissue microarray results in the current study. In summary, differences among studies may be due to different origins of tissues, tumor types, and analytical tools. From our assays, it can be inferred that Rab11-FIP2 is a tumor suppressor gene that participates in the initiation and/or progression of GC.

MiR-192/215 belong to the miR-192 family, possessing the same octameric seed sequence. In our previous study, we showed that miR-192/215 were upregulated in GC, consistent with their function as oncomiRs^9^. However, in CRC, it has been reported that miR-192/215 were significantly downregulated^27^. Khella et al. found that miR-192 and miR-215 formed a convergent miR network suppressing tumor progression in renal cell carcinoma^28^. Senanayake et al. showed that miR-192/215 are downregulated and target ACVR2B in renal childhood neoplasms^29^. Taken together, these results indicate that miR-192/215 can be regarded as either oncomiRs or tumor suppressor miRs, depending on tumor type. In our research, biological features of GC cells under the control of miR-192/215 via Rab11-FIP2 were examined. In summary, proliferation, invasion, and metastasis of GC cells were remarkably decreased by miR-192 /215 inhibitors. In contrast, these malignant behaviors were rescued by co-transfection with Rab11-FIP2 siRNA. Notably, this rescue effect was not as great for an miR-215 inhibitor. These results may have been due to other miR-215 target genes acting on the same phenotype. Our previous study revealed that ALCAM is a direct target of miR-192/215 in GC. Thus, miR-192/215 targets several genes during the development of GC, characteristic of other miRs in other cancers^9^.

We conclude that cancer-relevant biological functions of GC cells in vitro are modified by miR-192/215 via Rab11-FIP2. In our previous study, we also showed that miR-192/215 promoted proliferation in GC^9^. We also found that miR-194, another member of the miR-192/215 cluster, functioned as an oncogene in GC^30^. MiR-194 was overexpressed in GC cell lines and 43 paired GC tissues. Moreover, forced overexpression of miR-194 promoted cell proliferation and migration, while inhibition of miRNA-194 blocked these processes. Finally, inhibition of miRNA-194 decreased tumor volume in vivo^30^.

Rab11-FIP2 has been shown to control cell polarity. Recent evidence suggests that cell polarity proteins are targets of oncogenes, and an increasing number of tumor suppressors have been shown to regulate polarity pathways^20^. Epithelial cell polarity plays a tumor-suppressive role via participation in the establishment and maintenance of the organization of epithelial tissues as a whole^19^. Accumulating data have revealed that defects in cell polarity may result in tissue disorganization and subsequently lead to the initiation and progression of GC. For example, the tumor suppressors lgl, dlg, and Scrib have identical effects on epithelia and act together in a common pathway to regulate cell polarity and growth control. Dysregulation of Lgl2 correlates with GC progression^17,18^. However, there have been no previous studies addressing the relationship of Rab11-FIP2 to cell polarity in GC. To explore this relationship, the junction proteins α-catenin, β-catenin, and γ-catenin were evaluated in the current study. The loss of a key component of adherens junctions, E-cadherin, often occurs in later stages of tumorigenesis and is thought to contribute to EMT. Therefore, EMT proteins were also assessed in the current study. Results of these experiments suggested that regulation of Rab11-FIP2 by miR-192/215 exerts a tumor-suppressive effect in GC via cell junctions and EMT. Thus, our results suggest potential mechanisms underlying meaningful and potential significance of Rab11-FIP2 in gastric carcinogenesis.

In summary, we have discovered that RAB11-FIP2 is a target of oncomiRs miR-192/215, and that its downregulation is associated with GC progression. In addition, miR-192/215 regulates cell proliferation and migration in GC, at least in part, by participating in cell polarity, adherens junctions, and EMT via targeting of RAB11-FIP2.

## Materials and methods

### Cell lines and cell culture

HFE145 and BGC823 were cultured in DMEM medium supplemented with 10% fetal bovine serum in a 5% CO_2_ incubator at 37 °C. HFE145 was from Howard University (Dr. Duane T Smoot). BGC-823 was acquired from Cell Bank of the Chinese Academy of Sciences (Shanghai, China).

### Gene microarrays

To know the targets of miR-192/215, we performed gene microarrays, which were carried out by the Agilent Whole Genome Oligo Microarrays (4×44K, Agilent, Santa Clara, CA, USA). Different expression genes were identified between two groups of cells, BGC823 cells with inhibition of miR-192/215, and HFE145 cells with mimics of miR-192/215. Upregulated or downregulated at least two-fold genes were counted as significance. Briefly, TRIzol Reagent (Invitrogen, Carlsbad, California, USA) lyses cells, and RNA Integrity was tested by Agarose Gel Electrophoresis. Agilent Quick Amp Labeling Kit and Agilent’s SureHyb Hybridization Chambers (Agilent, Santa Clara, CA, USA) were applied for the sample labeling and hybridization as per the manufacture protocols. Data were extracted and further analysis was performed by Agilent GeneSpring GX 11.5.1 software.

### Real-time RT-PCR

mRNA expression was examined by real-time RT-PCR. Total RNA from the tissue samples and cultured cells was extracted using TRIzol Reagent (Invitrogen, Carlsbad, California, USA) according to established protocols. The Advantage RT-for-PCR Kit (Clontech, USA) and SYBR^®^ Premix Ex Taq^TM^ II (Takara, China) were used to synthesize cDNA and quantify the expression of Rab11-FIP2, respectively, which were all referred as the formal procedure^9^. GAPDH was used as the inner reference to normalize the mRNA expression. Primer sequences of Rab11-FIP2 were as given in the Supplementary Table 1. The relative mRNA levels were calculated using the ΔΔCtt method, which was done three times.

### Western blotting

The proteins extraction and Western blotting with Rab11-FIP2 (1:500, Sigma, USA), β-actin (1:2000, ZSBIO, CHINA), Adherens Junction kit (CST, USA), and EMT kit (CST, USA) antibodies were performed essentially as previously described^9^.

### Tissues and animals models

Fresh GC samples were obtained from patients prior to radiotherapy and chemotherapy at the Department of general surgery of the first Affiliated Hospital of Shenzhen University, Shenzhen, China. Tissues were saved immediately in RNAlater (Ambion, USA) after resection, and then stored at −80 °C until needed. For the use of these clinical materials for research purposes, patients' consent and approval from the Institute Research Ethics Committee were obtained.

To evaluate the tumor growth in vivo, animal models were set up, which was done following the procedure previously described^30^ in a specific pathogen-free environment. Subcutaneous tumor tissues were stained with Ki67 to test the proliferation abilities of the cells with different treatments. TUNEL assays were preformed as well to test the cell apoptosis in vivo. All the procedures followed the instructions of TUNEL (Promega). To evaluate metastasis in vivo, 5 × 10^5^ cells were injected into the tail veins of mice (*n* = 4 per group). After 8 weeks, the animals were euthanized, and the lungs were removed, rinsed, fixed, and subjected to pathological examination. Lung tissues were dissected and subjected to histological examination. Metastases were detected by H&E staining and quantified by counting metastatic lesions in each lung. Each group got an average number. All protocols for animal studies were reviewed and approved by the Institutional Animal Care and Use Committee of Medical College of Shenzhen University.

### Reagents and transfection

miR-192/215 mimics, inhibitors, and corresponding NC were obtained from Dharmacon (Lafayette, CO, USA). Cholesterol-conjugated miR-192/215 inhibitor and RAB11-FIP2 siRNA for in vivo RNA delivery, and their respective NCs were from Ribobio Co. (Guangzhou, China)^31^. When cells were filled with 50% confluence, we transfected miRs with 60 nM via Lipofectamine RNAi MAX (Invitrogen). Human Rab11-FIP2 siRNA (siRNA1, siRNA2, and siRNA3 sequences were listed in the Supplementary Table 1) were from Ribobio (Guangzhou, Ribobio, Co., Ltd). A scramble siRNA, which has no homology with the mammalian mRNA sequences, was used as control.

### Luciferase assay

For luciferase activity assay, Rab11-FIP2-3′UTR segments and Rab11-FIP2-3′UTR-mutant segments containing putative miR-192/215 binding site were inserted into psiCHECKTM-2 vector (Promega, USA). For the co-transfection of miR and luciferase report vectors, we transfected 60 nM miR and 40 ng of plasmids using Lipofectamine 3000 according to the instructions (Invitrogen, USA). The luciferase activity was measured 48 h after transfection by the Dual-Luciferase Reporter Assay Kit (Promega)^9^. Each assay was repeated in three independent experiments.

### Tissue microarrays and IHC

Tissue microarrays were collected from 40 cases tissues, including GC, para-cancer tissues, and corresponding lymph nodes. All the cases were attached with complete clinicopathology data for further analysis. Expression of Rab11-FIP2 was detected by IHC. The EnVision + detection system (Dako) was used following the manufacturer’s instructions. Primary anti-Rab11-FI2P antibody (Sigma, 1:50) was applied. The estimation of the stained tissues was performed by two pathologists in a blinded manner. The positive score of Rab11-FI2P was calculated as the sum of the percent positivity of stained tumor cells (“0”, 0%; “1”, 1–25%; “2”, 26–50%; “3”, 51–75%, and “4”, >75%) and the staining intensity (“0” means no staining, “1” means weakly stained, “2” means moderately stained, and “3” means strongly stained).

### Proliferation, plate colony formation, scratch assay, and cell invasion assays in vitro

#### Cell proliferation assay

Cells were seeded into 96-well plates and the cell numbers were counted after 0, 24, 48, and 72 h of incubation using Cell Counting Kit-8 Assay in triplicate. EdU proliferation assay (RiboBio Inc.) was carried out using the Cell-Light TM 5-ethynyl-20-deoxyuridine imaging detection kit (RiboBio) according to the manufacturer’s instructions. In brief, cells were seeded in 6 cm plates for 24 h; 24 h after transfection, the cells were incubated with 50 μM EdU for 5 h and fixed within 4% paraformaldehyde for 30 min at room temperature (RT). Cells were washed in PBS twice and permeabilized by 0.5% Triton X-100 for 10 min. Cells were incubated lucifugally in Apollo staining solution for 30 min. All the images were taken by confocal microscope.

#### Colony formation assay

Two-hundred cells were planted in each well of a 6-well plate. Two weeks later, the cell colonies were stained using Giemsa solution, and cultured for 2 weeks at 37 °C. The numbers of colonies per well were counted.

#### Scratch assay

For the scratch assay, gastric cells was cultured in a 6-well plate. When confluence was up to 90%, tips were used to scratch in the middle. At 24 and 36 h, images were taken under a microscope.

#### Cell invasion assay

Cell invasion assays were carried out using the Boyden invasion chambers to detect the invasion ability of cells. 10% FBS medium was added to the lower compartment and lower chamber as a chemoattractant; 1.5 × 10^5^ tumor cells were cultured in the upper compartment with serum-free medium. Twenty-four hours later, we counted the cells, which invaded through the chamber members, under the microscope. Each experiment was repeated three times.

### Statistical analysis

All statistical analyses were performed by SPSS 13.0 statistical software, which was considered significant when *p* < 0.05. Statistical significance among/between groups was tested using one-way ANOVA. The correlation between the expression of RAB11-FIP2 and various clinicopathological indexes was evaluated with *χ*^2^ test. Cell proliferation, cell cycle, and invasion assays in vitro were all tested by one-way ANOVA.

## Electronic supplementary material

Supplementary Figure1,Supplementary Figure2,Supplementary Figure3,Supplementary Figure4

Supplementary Table1, Supplementary Table2,Supplementary Table3

Supplementary figure legends
