# Supplementary material for: Inhibition of the miR-192/215–Rab11-FIP2 axis suppresses human gastric cancer progression
Source: Cell Death Dis. 2018 Jul 13;9(7):778. doi: 10.1038/s41419-018-0785-5 (PMC6045576; doi:10.1038/s41419-018-0785-5)
Supplement: Supplementary file 2 — Supplementary Table1, Supplementary Table2,Supplementary Table3 [file 41419_2018_785_MOESM2_ESM.pdf]

Supplementary Table 1. Rab11-FIP2 Primers and siRNA Sequences

	**Sequences of QPCR**
Rab11-FIP2F	5’CGGACAGCAACCCCTTTGA3’
Rab11-FIP2R	5’ GGCGTTTCTTCCATTACCCTTAC3’
**Sequences of siRNAs**	
Rab11-FIP2-siRNA1-F	5’CCGCAAGUAUGUUUGACUU DTDT 3‘
Rab11-FIP2-siRNA1-R	3’DTDT GGCGUUCAUACAAACUGAA 5‘
Rab11-FIP2-siRNA2-F	5’GCUACCUGGAUUGCUAAUU DTDT 3‘
Rab11-FIP2-siRNA2-R	3’DTDT CGAUGGACCUAACGAUUAA 5‘‘
Rab11-FIP2-siRNA3-F	5’GGAUGCAGUGAUUAAGGAA DTDT 3‘
Rab11-FIP2-siRNA3-R	3’DTDT CCUACGUCACUAAUUCCUU 5‘

Supplementary Table 2. Relationship between Rab11-FIP2 expression levels and clinicopathologic features in GC patients

Features	Total number	High expression	Low expression	λ^2	*P*
FIP2	40	2	38		
Age				0.005	0.942
<60	19	1	18		
≥60	21	1	20		
Gender				0.535	0.465
Male	29	1	28		
Female	11	1	10		
Tumor size				0.799	0.372
<5cm	11	0	11		
≥5cm	29	2	27		
Differentiation				0.234	0.89
Well	2	0	2		
Moderate	2	0	2		
Poor	36	2	34		
Lymphoma metastasis				2.167	0.141
N	11	0	11		
Y	29	5	24		

Supplementary Table 3. Target Genes Screened by Microarray

	Fold change and Regulation				Annotation	
**Cells**	**Fold change ([192i] vs [NSC])**	**Regulation**	**Fold change ([215i] vs [NSC])**	**Regulation**	**Genbank**	**GeneSymbol**
BGC823	2.50	up	2.32	up	NM_078628	MSL3
BGC823	4.52	up	2.00	up	NM_007076	FICD
BGC823	2.86	up	3.21	up	NM_014567	BCAR1
BGC823	**3.55**	**up**	**3.5**	**up**	**NM_014904**	**RAB11FIP2**
BGC823	3.94	up	2.13	up	NM_174942	GAS2L3
BGC823	3.18	up	2.77	up	NM_001402	EEF1A1
BGC823	1.92	up	1.87	up	NM_014795	ZEB2
BGC823	2.31	up	2.10	up	NR_002822	MGC72080
BGC823	2.37	up	2.35	up	NR_027046	LOC145474
BGC823	3.48	up	3.18	up	NM_003618	MAP4K3
**Cells**	**[192m] vs [NSC]**		**[215m] vs [NSC])**			
HFE145	2.44	down	2.41	down	NM_001134462	NOTO
HFE145	2.02	down	3.23	down	NM_016395	PTPLAD1
HFE145	3.32	down	2.10	down	NM_001702	BAI1
HFE145	3.50	down	2.21	down	NM_015035	ZHX3
HFE145	**3.08**	**down**	**5.07**	**down**	**NM_014904**	**RAB11FIP2**
HFE145	2.24	down	3.51	down	NM_032172	USP42
HFE145	2.12	down	1.95	down	NM_014795	ZEB2
HFE145	3.55	down	4.93	down	NM_174942	GAS2L3
HFE145	2.79	down	7.78	down	NR_027330	C7orf54
HFE145	2.59	down	2.74	down	NM_001042402	NAAA

Abbreviations:*192:*miR-192;*215:*miR-215;*i:*inhibitor;*m:*mimic;*NSC:*non-specific control
