# Supplementary material for: Inhibition of the miR-192/215–Rab11-FIP2 axis suppresses human gastric cancer progression
Source: Cell Death Dis. 2018 Jul 13;9(7):778. doi: 10.1038/s41419-018-0785-5 (PMC6045576; doi:10.1038/s41419-018-0785-5)
Supplement: Supplementary file 3 — Supplementary figure legends [file 41419_2018_785_MOESM3_ESM.docx]

**Supplementary Figure Legend**

**Supplementary Figure 1. Expression of miR -192 /215 in gastric cell lines.**

**Supplementary Figure 2. Expression of Rab11-FIP2 as a function of miR-192/215 regulation.** Left panel, Effects of miR-192/215 inhibitors and mimics on FIP2 expression (RT-PCR results). Right panel, Effect of Rab11-FIP2 siRNAs on FIP2 protein expression (Western blot results). NC: negative control; 192: miR-192; 215: miR-215; inh: inhibitor; mim: mimic.

**Supplementary Figure 3. Effects of miR-192/215 inhibitors and FIP2 siRNA on apoptosis (TUNEL results).**

NC: negative control; 192: miR-192; 215: miR-215; inh: inhibitor; mim: mimic; FIP2: Rab11-FIP2 siRNA.

**Supplementary Figure 4. Effects of miR and FIP2 inhibition on EMT protein levels.** EMT proteins ZO1 and claudin were measured by Western blotting. *NC:* negative control; *192:* miRNA-192; *215:* miRNA-215; *FIP2si:* Rab11-FIP2 siRNA; *mim:* mimic; *inh:* inhibitor
